# In situ hybridization to detect DNA amplification in extracellular vesicles

**DOI:** 10.1002/jev2.12251

**Published:** 2022-08-31

**Authors:** Lucia Casadei, Patricia Sarchet, Fernanda Costas C. de Faria, Federica Calore, Giovanni Nigita, Sayumi Tahara, Luciano Cascione, Martin Wabitsch, Francis J. Hornicek, Valerie Grignol, Carlo M. Croce, Raphael E. Pollock

**Affiliations:** ^1^ The Ohio State University Comprehensive Cancer Center Columbus Ohio USA; ^2^ Institute of Oncology Research (IOR), Faculty of Biomedical Sciences Università della Svizzera italiana (USI), Bellinzona, Switzerland, Swiss Institute of Bioinformatics (SIB) Lausanne Switzerland; ^3^ Department of Cancer Biology and Genetics The Ohio State University Columbus Ohio USA; ^4^ Department of Pediatrics and Adolescent Medicine Division of Paediatric Endocrinology and Diabetes Centre for Hormonal Disorders in Children and Adolescents Ulm University Hospital Ulm Germany; ^5^ Sarcoma Biology Laboratory, Department of Orthopaedics, Sylvester Comprehensive Cancer Center and the University of Miami Miller School of Medicine Miami Florida USA

**Keywords:** EV embedding, EV in situ hybridization, EVs, extracellular vesicles (EVs), FISH, sarcoma, tissue

## Abstract

EVs have emerged as an important component in tumour initiation, progression and metastasis. Although notable progresses have been made, the detection of EV cargoes remain significantly challenging for researchers to practically use; faster and more convenient methods are required to validate the EV cargoes, especially as biomarkers. Here we show, the possibility of examining embedded EVs as substrates to be used for detecting DNA amplification through ultrasensitive in situ hybridization (ISH). This methodology allows the visualization of DNA targets in a more direct manner, without time consuming optimization steps or particular expertise. Additionally, formalin‐fixed paraffin‐embedded (FFPE) blocks of EVs allows long‐term preservation of samples, permitting future studies. We report here: (i) the successful isolation of EVs from liposarcoma tissues; (ii) the EV embedding in FFPE blocks (iii) the successful selective, specific ultrasensitive ISH examination of EVs derived from tissues, cell line, and sera; (iv) and the detection of MDM2 DNA amplification in EVs from liposarcoma tissues, cell lines and sera. Ultrasensitive ISH on EVs would enable cargo study while the application of ISH to serum EVs, could represent a possible novel methodology for diagnostic confirmation. Modification of probes may enable researchers to detect targets and specific DNA alterations directly in tumour EVs, thereby facilitating detection, diagnosis, and improved understanding of tumour biology relevant to many cancer types.

## 1 INTRODUCTION

Soft tissue sarcomas (STS) are tumours of mesenchymal origin that can occur anywhere in the body. There are over 100 different histologic subtypes of STS: liposarcoma (LPS) is the most common subtype comprising 40% of STS. Liposarcomas are further divided into four different sub‐subtypes: well differentiated (WDLPS), dedifferentiated (DDLPS), myxoid, and pleomorphic based on histology and biologic factors. Of these, WDLPS and DDLPS (WD/DDLPS) are the most prevalent; when located in the retroperitoneum they can grow to massive size prior to clinical detection (retroperitoneal liposarcoma, RL). Surgical resection is the mainstay of treatment. For those that are unresectable or metastasize, systemic non‐specific multi‐drug chemotherapy regimens are mainstays but have modest efficacy for most LPS patients with overall response rates of only 20%–30% (Casadei et al., 2022). Radiotherapy also lacks overall survival impact. In addition to growing to massive size before detection, RL can also present or recur as synchronous multifocal lesions and are characterized by ultimately lethal multicentric recurrence in approximately 60% of patients (Tseng et al., 2021). The overall survival rate of 10% at 10 years has persisted for many decades. The lack of new effective treatments since the 1970 s, and the modest chemotherapy response rates, forces use of repeat radical surgery. At the molecular level, practically all WD/DDPLS RL have amplification of 12q13‐q22, the locus of MDM2, the most commonly overexpressed gene in this disease (Casadei & Pollock, 2020). Mechanistically, overexpressed MDM2 DNA leads to MDM2 protein overproduction that can override wtp53 tumour suppressor functions and is a key driver of RL progression. Diagnosis is generally confirmed by fluorescence in situ hybridization (FISH) for amplified MDM2 DNA in resected tissues. FISH uses fluorescent probes to detect DNA sequences; it is useful in identifying many types of chromosomal abnormalities in patient tissues (chromosomal rearrangements or gene aberrations), contributing to verification of diagnosis (Anaya et al., 2009; Kimura et al., 2013; Sugita & Hasegawa, 2017). Similarly, in situ hybridization (ISH) is a cytogenetic technique allowing detection, quantification, and localization of nucleic acid targets inside cells or tissues. This method is based on the hybridization of sequence‐specific complementary probes (typically DNA sequences) to their targets inside the cell (Huber et al., 2018). The sensitivity of ISH methods has remained a concern, and methods aimed at enhancing ISH sensitivity have been developed over the years (Andras et al., 2001; Qian & Lloyd, 2003). Among some of the newest approaches, scope methodologies (DNA/RNAScope, Biotechne, Canada) are a commercially available ultrasensitive ISH assay based on branched or ‘tree’ methodology (Wang et al., 2012). The advantage of this method is its sensitivity that allows single molecule detection (Player et al., 2001; Wang et al., 2012). DNAscope chromogenic duplex (red/blue) staining allows researchers to use a standard bright‐field microscope to visualize and quantify gene copy number variations (amplifications/deletions) and gene rearrangements/fusions within tissues. It is particularly sensitive due to its characteristic amplification of target‐specific signals (https://acdbio.com/dnascope‐duplex‐assay).

Extracellular vesicles (EVs) are secreted lipid bilayer‐enclosing vesicles (Lötvall et al., 2014). Given the emerging awareness of EVs in communication between tumour and microenvironment, it is pertinent that studies have shown a possible role for EV cargoes in cancer initiation, progression and metastasis (Bhatta & Cooks, 2020; Guo et al., 2019; Urabe et al., 2021). EV cargos as proteins, mRNA and miRNAs have been studied extensively as potential biomarkers for cancer diagnosis and prognosis (Chen et al., 2018; Huang & Deng, 2019); for example, EV miR‐150, miR‐155 and miR‐29 family members in chronic lymphocytic leukaemia, EV miR‐19a, miR‐19b, miR‐23a, miR‐92 a, miR‐320a and miR‐4437 in colorectal cancer; miR‐1246, miR‐718 in hepatocellular carcinoma; EV miR‐127‐3p, miR‐155‐5p, miR‐21‐5p, miR‐24‐3p, and let‐7a‐5p, in classical Hodgkin lymphoma (Drees et al., 2021). Protein signatures of EVs have high accuracy in discriminating metastatic from non‐metastatic breast cancer (Tian et al., 2021); for a detailed review see Kinoshita et al. (2017). Several investigators have reported that human cancer cell line‐derived EVs can transform non‐malignant, non‐tumour cells in experimental systems in vitro and in vivo; for example, CRC cell line‐derived EVs transform patient‐derived stromal cells (Lugini et al., 2016) and prostate cancer cell‐derived EVs transform patient‐derived adipose stem cells (Abd Elmageed et al., 2014; Xu et al., 2018).

Information on the role of EVs in sarcoma is currently limited. EVs in liposarcoma have been described by our group (Casadei & Pollock, 2020; Casadei et al., 2019, 2017, 2021). Major cargos in liposarcoma EVs include MDM2 DNA (Casadei et al., 2019) and specific miRNAs (miR‐25‐3p, mir‐92a‐3p (Casadei et al., 2017)). RL EV cargo has been described to play an important role inducing tumour progression and metastasis (Casadei & Pollock, 2020; Casadei et al., 2019, 2022) Specifically, it has been shown that the transfer of EV‐derived MDM2 DNA from RL to preadipocytes in the RL microenvironment promotes production of metalloproteinases (specifically MMP2) which could lead to pre‐metastatic niche preparation and subsequent recurrences in RL.

Recently, interest has been growing regarding the possible roles of EV cargo DNA (Malkin & Bratman, 2020). The presence of DNA in EVs was first described in studies by Thakur et al. and Kahlert et al. in 2014, where the presence of double strand genomic DNA was reported in EVs and it was found that EV‐derived DNA reflected the genetic aberrations of the tumour of origin (Kahlert et al., 2014; Thakur et al., 2014; Vagner et al., 2018). Mitochondrial DNA has also been found in EVs (Guescini et al., 2010), and the effect of mtDNA transfer as an oncogenic signal has been investigated (Sansone et al., 2017). Since its discovery, EV DNA has been implicated in physiological functions such as embryogenesis, fetal‐maternal crosstalk, and thrombosis (Malkin & Bratman, 2020). EV DNA has also been implicated in disease processes such as tumorigenesis and the development of the pre‐metastatic niche (Casadei et al., 2019; Li et al., 2019; Wortzel et al., 2019). Since EV DNA reflects the parental cell genomic DNA, both qualitatively (Allenson et al., 2017; García‐Romero et al., 2017; Yang et al., 2017) and quantitatively (Guescini et al., 2010), it has been reported that the analysis of circulating EV DNA may have substantial diagnostic potential. Moreover, analysis of genomic mutations has been hypothesized to be advantageous compared to analysis of EV RNA because DNA is intrinsically more stable than RNA (Gézsi et al., 2019).

Many studies have focused on EVs isolated from cell line conditioned media (CM); however, this in vitro system has many limitations: EVs isolated from CM may be affected by high numbers of passages as part of their long‐term cultivation (Ramirez et al., 2018), and EVs isolated form these cell lines may therefore have altered characteristics (Allen et al., 2016; Crescitelli et al., 2021). Moreover, cells in culture can lose the influence of other TME cells. Therefore, EVs isolated directly from tissues may be more informative; however, studies of EV isolated from tissues remain limited (Huang et al., 2020; Hurwitz et al., 2019) paraphs because of difficulties in obtaining contaminant‐free pure EV preparations (Crescitelli et al., 2021). Studying EVs secreted directly from tissues is a new concept (Zieren et al., 2020). In 2017, Vella et al. isolated EVs from human brain tissue (Vella et al., 2017). More recently, Jang et al. studied EVs from human melanoma and Jingushi et al. isolated EVs from human kidney tumours (Jang et al., 2019; Jingushi et al., 2018). Isolation of EV subpopulations from melanoma and colon cancer tissues was achieved by Crescitelli et al. (2021). Protocols for EVs isolation is critical for consistent results, and variations in EV isolation protocols may lead to discordant results (Coumans et al., 2017; Théry et al., 2018). Compared to methods used for EV isolation from CM, methods for EV isolation from tissues still needs to be optimized.

Interest in image EVs is receiving attention; VerweiJ et al. recently suggested that solving technical pitfalls hindering EV imaging might help to elucidate the structure and function of EVs in normal and diseased states and understanding EV biology (Verweij et al., 2021), a prospective also suggested by Gupta et al. (2019). VerweiJ et al. also discussed how advances in imaging could address open questions in EV biology, from biogenesis to uptake and function, thereby enhancing the development of EV therapeutics (Verweij et al., 2021). In recent years, EV labelling strategies have been developed to allow visualization or separation of EVs based on their surface proteins or other characteristics, for example, lipid dyes versus, luminal dyes or genetic labelling (Verweij et al., 2021). Techniques for the detection of EV proteins, mRNA and miRNA have also recently been developed (Ahmed et al., 2018; Fang et al., 2021; Gupta et al., 2019; Han et al., 2021; Lee et al., 2018, 2015; Wang et al., 2020). Gupta et al. have established a methodology for visualization of EVs using non‐reversible tissue fixation (Gupta et al., 2019).

We wanted to develop a simple, time saving, low‐cost methodology to embed EVs isolated from CM, tissues, and serum. In our previous work studying EV isolated from cell lines and serum, we showed that EVs released from RL contain MDM2 DNA cargo at amplified levels compared to normal controls. Because of this specific RL characteristic, we then sought to visualize MDM2 in EVs embedded using ultrasensitive ISH and a specific probe to detect this DNA.

## 2 MATERIALS AND METHODS

### 2.1 Patients

Retroperitoneal liposarcoma tumour tissues (*N* = 6) and adjacent healthy tissues (*N* = 3) were collected on an IRB‐approved tissue banking protocol at The Ohio State University Wexner Medical Center James Cancer Hospital. The detailed characteristics of patients and healthy controls participants are summarized in Supplementary Table 1. Serum samples of RL patients (*N* = 23) were also collected prior to surgery. Patient venous blood (12 ml) was collected in Vacutainer Plus whole blood tubes. Serum was retrieved from the whole blood samples via centrifugation at 1900 × *g* × 10 min at 4°C, then aliquoted and stored at −80°C until analysis. Healthy donor blood (*N* = 15) was purchased from ZenBio (Durham, NC). The detailed characteristics of patients and healthy controls participants are summarized in Supplementary Tables 2 and S3. Prior to any evaluation, patient pathology was confirmed using surgically resected sarcomas which were graded as per standard FNCLCC criteria. Samples were collected as per OSU IRB protocol number 2014C0028, approved in 2014. Patients consented as per protocol prior to any specimen collection.

### 2.2 Cell culture

Human liposarcoma (LPS) cell lines Lipo246 was established in our laboratory as previously reported (Peng et al., 2011). Cells were maintained using standard conditions and were grown in DMEM (Gibco), supplemented with 10% (vol/vol) FBS. SGBS P‐a were cultured in DMEM/Ham's F12 (1:1) containing 33 μmol/L biotin, 17 μmol/L pantothenate, antibiotics (serum‐free, basal medium), and 10% FBS.

### 2.3 EV isolation from RL tissues

**FIGURE 1:**
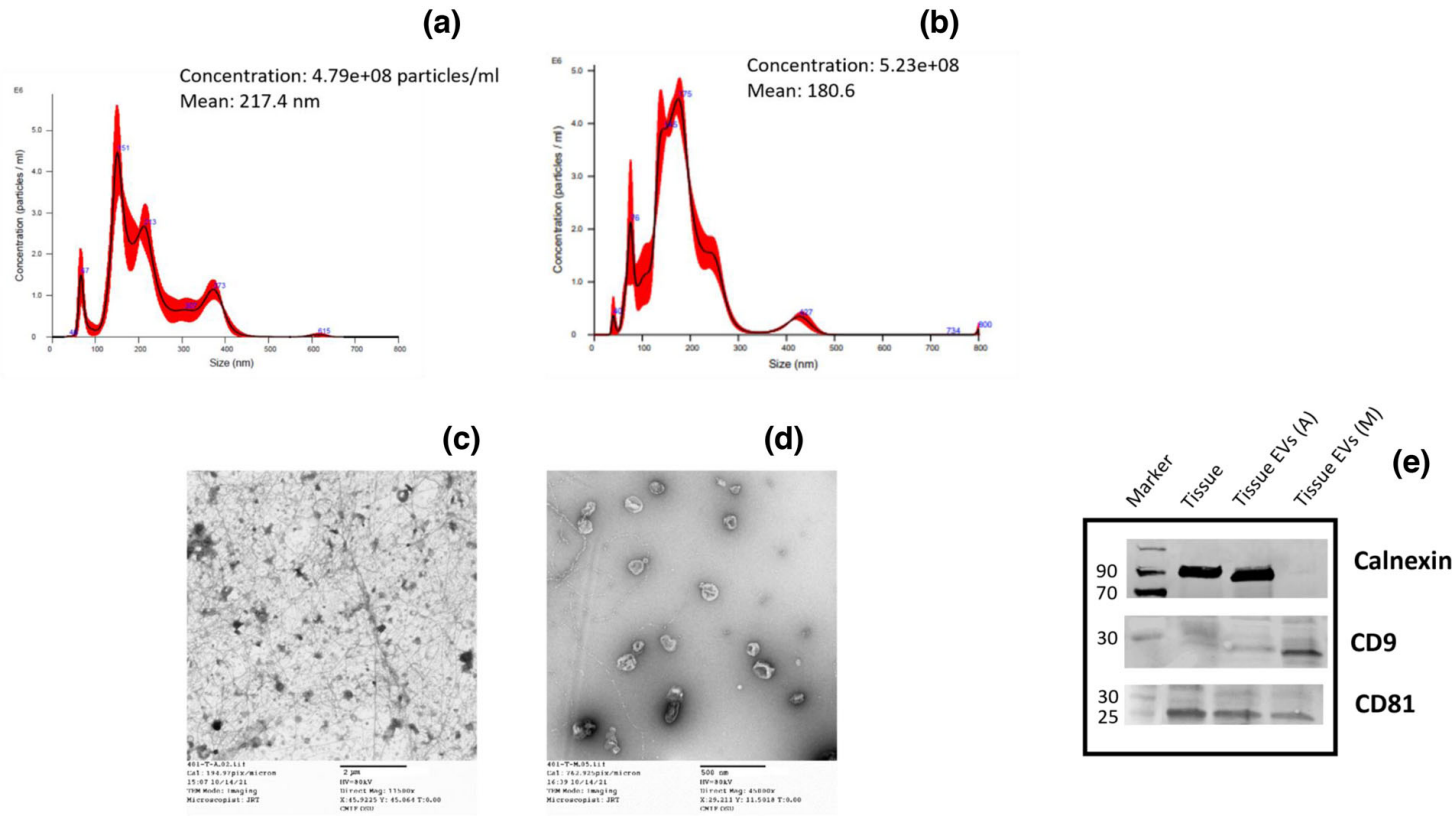
RL Tissue‐EV Characterization. Nanotrack analysis (Nanosight) on EVs isolated from the same RL tissue using method A (a) or method M (b). TEM analysis on EVs isolated from the same RL tissue using method A (c) or method M (d). WB analysis on EV isolated from the same RL tissue using methos A or method M (e). Representative images

Tissues resected from RL patients were weighted and 1 g was used for the EV isolation using two different methodologies namely method M and A, as described in Supplementary Figure 1. In method M, we used the Miltenyi Biotech tumour dissociation kit (human) in association with the gentleMACS Octo Dissociator (Miltenyi Biotech, Auburn, CA) with heater following the dissociation kit protocol. Briefly, 1 g of tissue was slightly cut and placed in specific tubes together with DMEM and H, R, A enzymes (provided by Miltenyi). Then the tube was placed in the gentle MACS octo dissociator with heater at 37°C for 61 min (following the Gentle MACS Program: 37c_h_tdk_1). In an alternative dissociation method, method A, we followed the dissociation method as described in Crescitelli et al. (2021). Briefly, tumour tissue was cut into small pieces placed in a six well plate together with appropriated collagenase and DNAse and incubated, in a shaking incubator at 37°C for 30 min. Once tumour was dissociated using procedure A or M, the cell suspensions were filtered through a 100 μM filter and then serial centrifugation (as for EV isolation from conditioned medium ‐CM‐ and sera) and ultracentrifugation were performed, as previously described (Casadei et al., 2019, 2017). The EV size assessment was performed by Nanotrack analysis (Nanosight, Figure 1a, b and Supplementary Table 4), the quality of isolated particles was assessed through TEM (Figure 1c, d), while we verified the purity of isolated particles by Western blot (Figure 1e).

### 2.4 EV isolation from CM and serum

**FIGURE 2:**
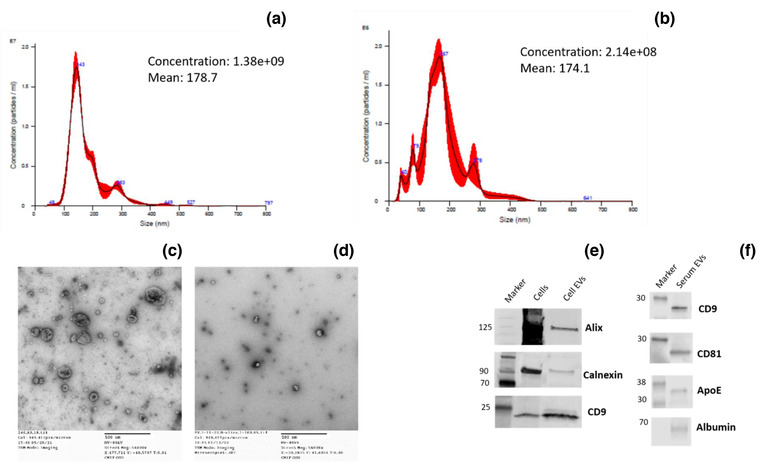
EV characterization from RL cell lines and sera. Nanotrack analysis (Nanosight, a) and TEM (c) for EVs isolated from RL cell line (Lipo246); Nanosight (b), TEM (d) for EVs isolated from serum. WB showing presence of Alix, CD9 and low level/absence of Calnexin in the EVs isolated form cell line (e), WB showing presence of CD9, CD81 and low level of ApoE and Albumin in the EVs isolated from sera (f)

EVs were isolated from Lipo246 CM and serum as previously reported (Casadei et al., 2019, 2017; Casadei et al., 2021). Briefly, EVs were isolated from 1.25 × 10^8^ cells cultured in serum‐free medium for 48 h. Next, serum‐free conditioned media were collected and harvested at 300 × *g* for 10 min, in order to eliminate large cells. The supernatant was carefully recovered, transferred to new, clean tubes and further centrifuged at 2000 × *g* for 20 min in order to remove dead cells. Once carefully transferred to new tubes, the supernatant was then centrifuged at 10,000 × *g* for 30 min in order to eliminate cell debris. Finally, one ultracentrifuge at 100,000 × *g* was performed for 70 min in order to pellet EVs. The resulting pellet was then washed in PBS and a second ultracentrifugation was performed at the same speed. As for EV isolation from sera, 5 ml serum was serially centrifuged at 300 × *g* for 10 min, 2000 × *g* for 20 min, 10,000 × *g* for 30 min. Then ultracentrifuge at 100,000 × *g* was performed for 70 min; the pellet was washed in PBS and a second ultracentrifuge was performed at the same speed. The EV pellet derived from both CM and sera was used for analysis and EV characterization. The EV size was determined by nanotrack analysis (Nanosight, Figure 2a, b), the quality of isolated particles was assessed through TEM (Figure 2c, d), while we verified the purity of isolated particles by Western blot (Figure 2e, f).

### 2.5 EV embedding

After EVs isolation from tissues, serum or cell CM, a thin layer of liquefied agarose (1% agarose: NuSieve GTG Agarose, Lonza) was spread out over the surface of a 6 well plate. The EV pellet was then placed in the liquid agarose. A thin layer of liquid agarose was placed over the EV pellet and allowed to solidify at 4°C. Solid agarose/EV pellet was removed from the plate, placed in a tissue cassette and fixed in 4% paraformaldehyde (PFA) overnight before embedding. FFPE blocks were then cut and mounted onto slides.

### 2.6 EV DNAScope

EV slides were probed for MDM2/CEP12 adapting the methodology for the DNAscope HD Duplex detection kit protocol for tissue. DNAscope has been designed only for the detection of DNA specifically in the slides of paraffin embedded tissues. Instead, we isolated EVs from the tissue, embedded them and use DNAscope on the tissue EVs. Slides were baked for 1 h at 60°C then deparaffinized as follows: slides were first incubated in fresh xylene for 5 min, then moved to another fresh container of xylene and incubated for another 5 min; then immediately placed in 100% ethanol for 2 min and in another dish of fresh 100% ethanol for another 2 min. Slides were then dried for 5 min at 60°C. A barrier was drawn around the tissue section using an immedge hydrophobic barrier pen. RNA removal solution was added to the slides, and they were incubated at 40°C in the humidity oven for 30 min. Slides were then incubated 10 min at RT with Hydrogen peroxide and at 40°C in the humidity oven for 15 min with protease plus reagent. diH_2_O and DNAscope Target retrieval reagent was warmed to 99°C using a steamer and the slides were acclimated in diH_2_O for 10 s prior to being placed in the prewarmed target retrieval reagent for 30–45 min. After removal from the target retrieval reagent and rinsed with hot diH_2_O, the slides were then incubated at 40°C in the humidity oven for 15–18 h with MDM2 and CEP12 probes. Slides were successively washed 2× at RT with 1× wash buffer and then incubated with AMP 1 at 40°C in the humidity oven for 30 min. This same step has been repeated with AMPs 2, 3, 4. Slides were washed 2× at RT with 1× wash buffer, AMP 5 was added to the slides and then slides were incubated at RT for 15 min. Slides were washed 2× at RT with 1× wash buffer and then the red signal was detected by adding a ratio of 1:50 red‐b to red‐a to the slides and allowed them to incubate at RT for 10 min. Slides were washed 2× at RT with 1× wash buffer and then AMP 6 was added to the slides, and they were incubated at 40°C in the humidity oven for 15 min. This same step has been repeated with AMP7, AMP8, AMP9, AMP10. Slides were washed 2× at RT with 1× wash buffer and then the blue signal was detected by adding a ratio of 1:50 blue‐b to blue‐a to the slides and allowed them to incubate at RT for 10 min. Slides were washed 2× at RT with 1× wash buffer. The slides were rinsed in diH_2_O and incubated 2 min at RT in 50% gill's hematoxylin 1. Slides were rinsed with diH_2_O, then rinsed with 0.02% ammonia water 5× and finally with diH_2_O and dried at 60°C for 10–15 min. Slides were dipped in fresh xylene and then vectamount was placed on the slides and they were cover slipped. Slides were allowed to dry prior to imaging.

### 2.7 Image analysis method

DNAscope images were analysed using Colour Deconvolution plugin from the FIJI‐ImageJ program, as described in Morley‐Bunker et al. (2021). Briefly, colour deconvolution was applied at images by choosing ‘From ROI’ vector, followed by selection of three different colours: Colour 1: vesicle; colour 2: MDM2 probe; Colour 3: Background. Three colour images were generated and MDM2 probe image (colour 2) was used for quantification by applying Otsu method threshold. MDM2 probe dots were then measured using the ‘analyse particles’ from FIJI‐ImageJ menu. In parallel, manual counting has been also performed from two different operators and compared to Image J detection for accuracy. The mean of two or three different pictures from each slide and of Image J and manual counting of two different operators has been reported. The mean number of dots has been normalized to the number of EVs present in the slides, determined by IF.

### 2.8 DNA isolation, Q‐PCR and molecular number variation

Total DNA derived from tissues, cell lines, and EVs was isolated by using the Qiagen kit following the manufacturer's protocol. The expression level of MDM2 starting from a DNA preparation (cell line, tissue and tissue‐EVs) was determined using DNA sequence–specific probes (MDM2‐ Hs00540450_s1, Thermo Fisher). For real time PCR on tissues and tissue‐EVs, the results were normalized on GAPDH (Hs03929097_g1, Thermo Fisher) after equal quantity (gM) of starting tissue, and equal input of DNA in Q‐PCR were used. As for Q‐PCR of MDM2‐DNA from cellular EVs the same number of cells and/or the same EV quantity has been used and verified by Nanosight (as previously described, Casadei et al., 2019). For the Q‐PCR on the DNA EVs of the serum, the normalization was volumetrically performed (as for Casadei et al., 2019, 2017). Determination of the number of molecules of MDM2 in the cellular EVs and serum EVs was performed using standard curve methodology following the methodology reported in Casadei et al. (2019). First, we performed serial dilution of MDM2 synthetic oligo and calculated the number of molecules of MDM2 that corresponded to each different concentration. Then, a RT‐PCR using the MDM2 probe (MDM2‐ Hs00540450_s1, Thermo Fisher) was performed using these diluted synthetic oligo samples (Integrated DNA Technologies). A standard curve was then performed in which a specific number of molecules was assigned based on the corresponding *C*_t_ value. All samples were run in duplicate or triplicate.

### 2.9 TEM

Samples were fixed with 0.5% glutaraldehyde in 0.1 M phosphate buffer (pH 7.4). EVs were adsorbed onto formvar‐carbon coated grids (EMS) by glow discharging (Pelco EasiGlow) grids and then incubating 7.5 ul of sample on the grids for 20 min at room temperature. Samples were fixed with 1% glutaraldehyde, stained with 1% uranyl acetate and imaged with an FEI Technai G2 Spirit transmission electron microscope (FEI), Macrofire (Optronics) digital camera, and AMT image capture software.

#### 2.9.1 Immunofluorescence (IF)

Slides were deparaffinized using Xylen and EtOH, then treated with citrate buffer 20 min at 95°C. The slides were then rinsed in 1× phosphate buffered saline (PBS) before blocking with 1% normal donkey serum (Jackson) and 0.3% Triton X‐100 in PBS for 1 h at room temperature. Slides were incubated with primary antibodies overnight at 4°C in blocking solution (Anti‐CD9 antibody; Abcam #ab82390 and anti‐CD81 antibody; Thermofisher #MA5‐13548). The next day, slides were washed with PBS and incubated with fluorescently conjugated secondary antibodies diluted in blocking solution for 2 h at room temperature. After washing, EVs were cover slipped with permanent mounting medium (VectaMount) before imaging on a Zeiss Axio Observer Z.1. The quantity of EVs present in the slides, were estimated counting the CD81 fluorescent signals.

#### 2.9.2 Nanotrack analysis (Nanosight)

All samples were diluted in PBS to a final volume of 1 ml. Pre‐testing has been done to assess the ideal measurement concentration. The settings were set according to the manufacturer's software manual (NanoSight NS300 User Manual, MAN0541‐01‐EN‐00, 2017). The detection threshold was determined to include as many particles as possible with the restrictions that 10–100 red crosses were counted, and only <10% were not associated with distinct particles.

### 2.10 Western blotting

For immunoblotting analysis, cells were lysed with ice‐cold NP‐40 Cell Lysis Buffer (Invitrogen) supplemented with protease inhibitors (Roche) for 30 min at 4°C. Equivalent amounts of protein were first mixed with sample buffer, then loaded on a Criterion Tris‐HCl 4%–20% precast gel (Bio‐Rad) and transferred to PVDF or nitrocellulose membranes. Membranes were incubated overnight at 4°C with commercially available antibodies as indicated per experiment: Alix (#SAB4200477, Sigma) calnexin (#C7617, Sigma); CD9 (#D8O1A, Cell Signalling Technology); CD81 (#10630D, Invitrogen), ApoE (#sc‐390925, Santa Cruz), Albumin (#A6684, Sigma). The proteins of interest were detected through chemiluminescence reaction.

### 2.11 Statistical analysis

Two‐tailed Student's *t*‐test was applied for Figures 4, 5, 6c, while Wilcoxon rank sum exact test was used for Figures 4f and 6d. This choice was based on Shapiro‐Wilk test to estimate the normality distribution on the data in input. Student's *t*‐test, Wilcoxon rank sum exact test and Shapiro‐Wilk test were performed by using *t*‐test, Student's *t*‐test and Wilcoxon rank sum exact test were performed by using *t*‐test, Wilcox‐test and Shapiro‐test functions, respectively, from stats R package (v4.1.1). Spearman correlation analyses were performed by employing cor‐test function from stats R (v.4.1.1) package.

## 3 RESULTS

### 3.1 In situ hybridization of tissue EVs

Two separate dissociation methods have been used to attempt the isolation of EVs from RL tissues. These methods will be referred to as: M and A. Method A has been adapted from Crescitelli et al. (2021). In method M, the Miltenyi Biotech tumour dissociation kit and protocol was used. The detailed steps of the EV isolation procedure have been summarized in Supplementary Figure 1 and described in the materials and methods section. We then compared the size, yield and purity of the EVs isolated by either method. The EV size and yield were assessed using nanotrack analysis (Nanosight). EVs of average sizes 209.1 nm and 200.5 nm were isolated respectively as per method M and A (Supplementary Table 4); representative images of nanotrack analysis for tissue EVs isolated by method A and M are shown in Figure 1a, b. TEM analysis has been used to determine the presence of EVs and purity of the preparations. As shown in Figure 1c, Method A resulted in an EV preparation containing more debris compared to Method M (Figure 1d). We then verified the presence of typical EV proteins (e.g., CD9, CD81) and the absence of cellular contaminants (e.g., Calnexin) in these preparations. Figure 1e shows WB performed on EVs isolated using the methods A and M. While the CD81 marker of EVs was present in both preparations, the marker CD9 was present only in the EVs isolated by method M. Furthermore, the method A had more cellular contaminants (presence of Calnexin) compared to method M (Figure 1e).

**FIGURE 3:**
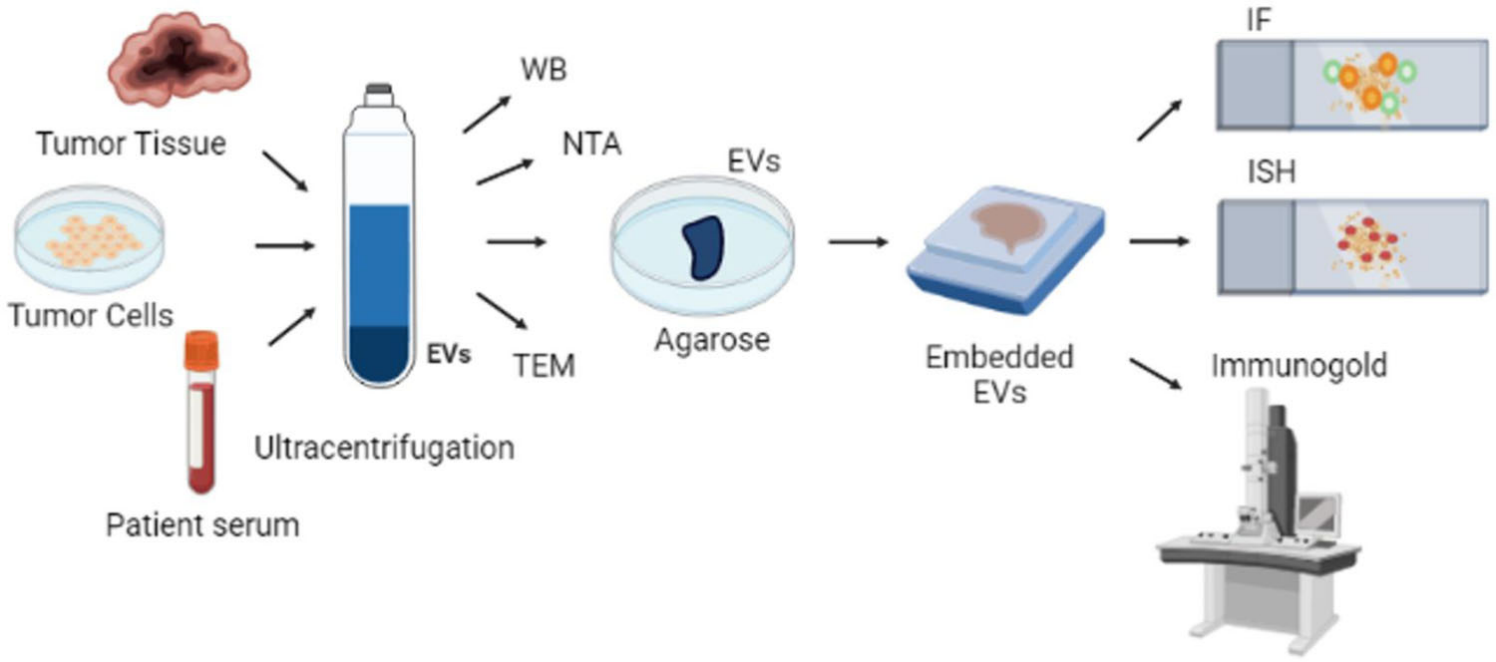
EV embedding and probing. EVs were isolated from tissues, sera or cell CM by ultracentrifugation, then characterized by transmission electron microscopy (TEM), nanoparticle tracking analysis (NTA) and western blot (WB) and placed in a thin layer of liquefied agarose covered with liquid agarose. Agarose/EV pellet was then placed in a tissue cassette and fixed in 4% parafolmaldehyde overnight before embedding. FFPE blocks were then cut and mounted onto slides. Slides were used for immunofluorescene (IF), ultrasensitive in situ hybridization (ISH), and immunogold TEM

Having established the elective method for EV isolation from RL tissues, EVs were then isolated from RL from equivalent amounts (1 gM) of tumour and normal tissues using method M, followed by serial centrifugations and ultracentrifugation (as described in materials and methods). The resultant pellets were divided for EV characterization and for formalin‐fixed paraffin‐embedded (FFPE) blocks using a system described in materials and methods, summarized in Figure 3. The patient characteristics are described in Supplementary Table 1. Once EVs were isolated from tissues, embedded slides were made as described in materials and methods. To verify the presence of EVs in the obtained block, we deparaffinized the blocks, extracted the EVs covered in agarose, and analysed the EVs by TEM. Supplementary Figure 2a shows that EVs are present in the embedded blocks.

**FIGURE 4:**
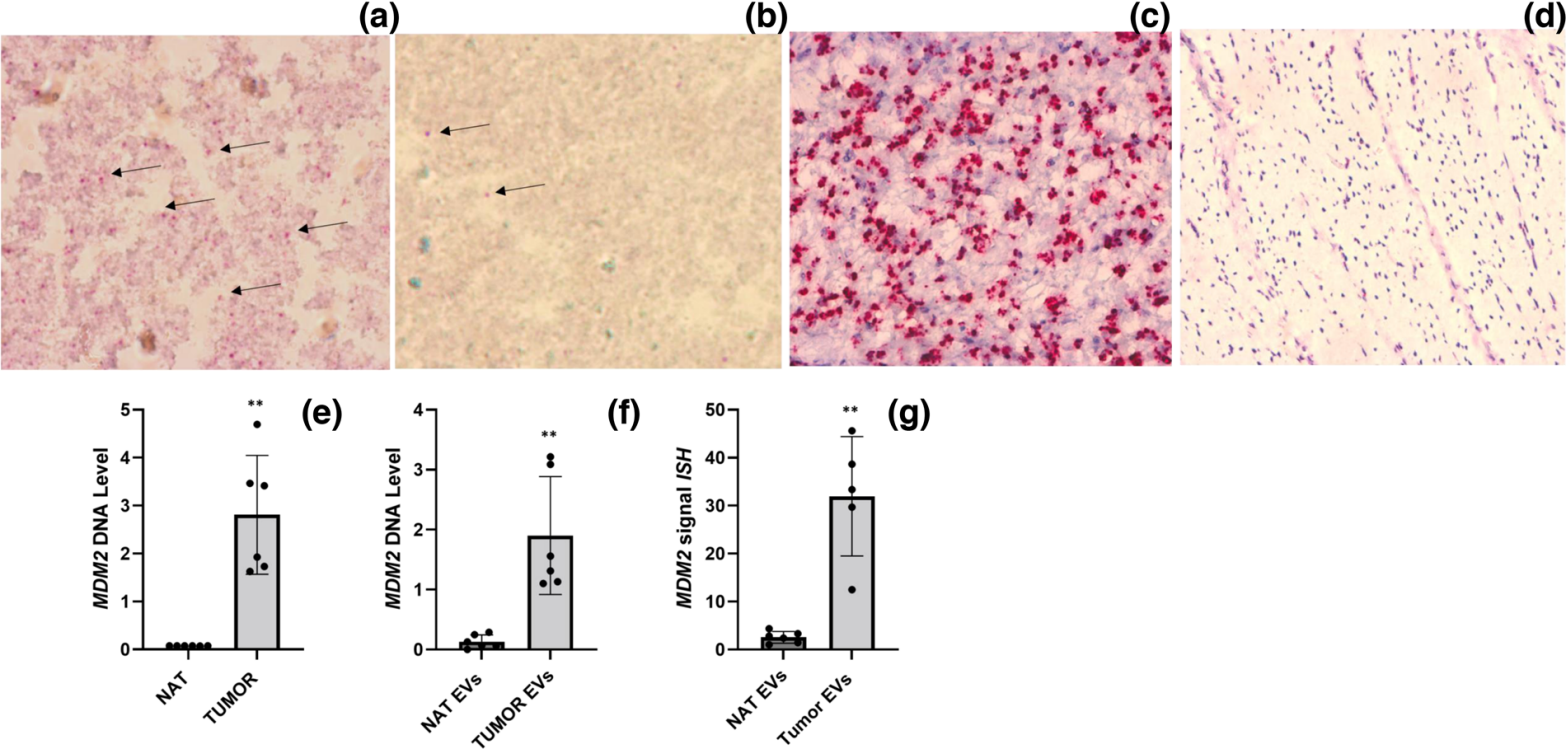
In Situ Hybridization on Tissue EVs. ISH for MDM2/CEP12 on EVs isolated from RL tissues (a); ISH for MDM2/CEP12 on EVs isolated from NAT (b). ISH for MDM2/CEP12 on RL tissues (c); ISH for MDM2/CEP12 on NAT (d), representative images. Level of MDM2 DNA in the same tissues used for ISH, RL tissues versus NAT (Q‐PCR) (e); Level of MDM2 DNA in EV isolated from the same tissues used for ISH, RL tissue EVs versus NAT EVs (Q‐PCR) (f). ISH signal quantification of MDM2 in RL‐EVs versus NAT‐EVs (g). MDM2 DNA: pink; CEP12: blue. Results are presented as average ± SD. Statistical analyses were performed using an unpaired t test; Wilcoxon rank sum exact test was used for Figure 4f. **, 0.001 ≤ *P* ≤ 0.01; ***, *P* ≤ 0.001

Sections of EVs in the slides were probed for MDM2/CEP12, adapting a methodology from DNAscope (chromogenic duplex red/blue, materials and methods). After probing, the presence of MDM2 (pink/red dots) was detected in the EVs isolated from the RL tissues (Figure 4a), demonstrating the possibility of probing for a sequence of DNA in EV preparations isolated from tissues. When we probed for MDM2/CEP12 in the EV isolated from Normal Adjacent Tissue (NAT), the MDM2 probe signal was extremely low compared to the MDM2 signal in the EVs from RL tissues (Figure 4b). To confirm the presence of high level of MDM2 in the tissue itself, we then probed for MDM2/CEP12 in the tissues from which the EVs had been isolated. As expected (Figure 4c), the tumour tissues revealed the presence of MDM2 DNA (visualized as pink/red dots) and the presence of the CEP12 control (blue dots), whereas the normal tissues showed only the signal corresponding to the CEP12 probe (Figure 4d).

To assess the specificity of the results obtained by ISH, Q‐PCR for MDM2 DNA was performed on the same ISH‐analysed tissues and their corresponding EV preparations. As shown in Figure 4e and f, MDM2 was highly expressed in both the tissues and tissue associated EVs analysed when compared to NAT and NAT EV (mean: NAT = 0.076 ± sd 0.0009, RL tumour = 2.809 ± sd 1.240, *p* = 0.0029; NAT EVs 0.13 ± sd 0.11, RL tumour EVs = 1.9 ± sd 0.98; *p* = 0.002). We then quantified the signal of MDM2 in the EVs isolated from different RL tumours and compared this signal to that of paired NAT EV for these same tumours using ImageJ software (as described in Materials and methods). Since there is uncertainty about the presence of stable and constant DNAs in the EVs, we could not use an internal DNA as normalizer. The signal of MDM2 was normalized to the quantity of EVs present in the slides. This quantity has been determined by IF and representative images are shown in Supplementary Figure 3. As shown in Figure 4g, the normalized MDM2 signal was amplified in the EVs isolated from RL tumour compared to those isolated from paired NAT (mean MDM2 signal ISH: NAT EVs = 2.55 ± sd 1.23, tumour EVs = 31.96 ± sd 12.4; *p* = 0.0059). Finally, to demonstrate the accuracy of probing EV DNA amplification by ISH in tissue EV samples, correlation analysis between MDM2 signals measured by ISH and Q‐PCR in the same patient cohort has been performed using Spearman's rank correlation test: Spearman's correlation coefficient Rho = 0.845, *p* = 0.002.

### 3.2 Ultrasensitive in situ hybridization of cell EVs

EVs were isolated from RL Lipo 246 and control P‐a (SGBS) cell lines using serial centrifugations followed by ultracentrifugation (as described in Materials and methods). The resultant pellets were divided for both EV characterization and embedding using the system described in materials and methods and summarized in Figure 3. EV characterization (size and quantification) was determined by nanotrack analysis (Nanosight) as well as TEM and WB for characteristic markers (Alix, CD9, Calnexin) as shown in Figure 2a, c, e. Characterization of Lipo246 EV has also been previously reported by us (Casadei et al., 2019, 2017). EVs were then embedded, and slides prepared as described for tissue EVs above. To verify the presence of EVs in the obtained block, we deparaffinized the blocks, extracted the EVs covered in agarose, and analysed the EVs by TEM. Supplementary Figure 2b shows that EVs are present in the embedded blocks.

**FIGURE 5:**
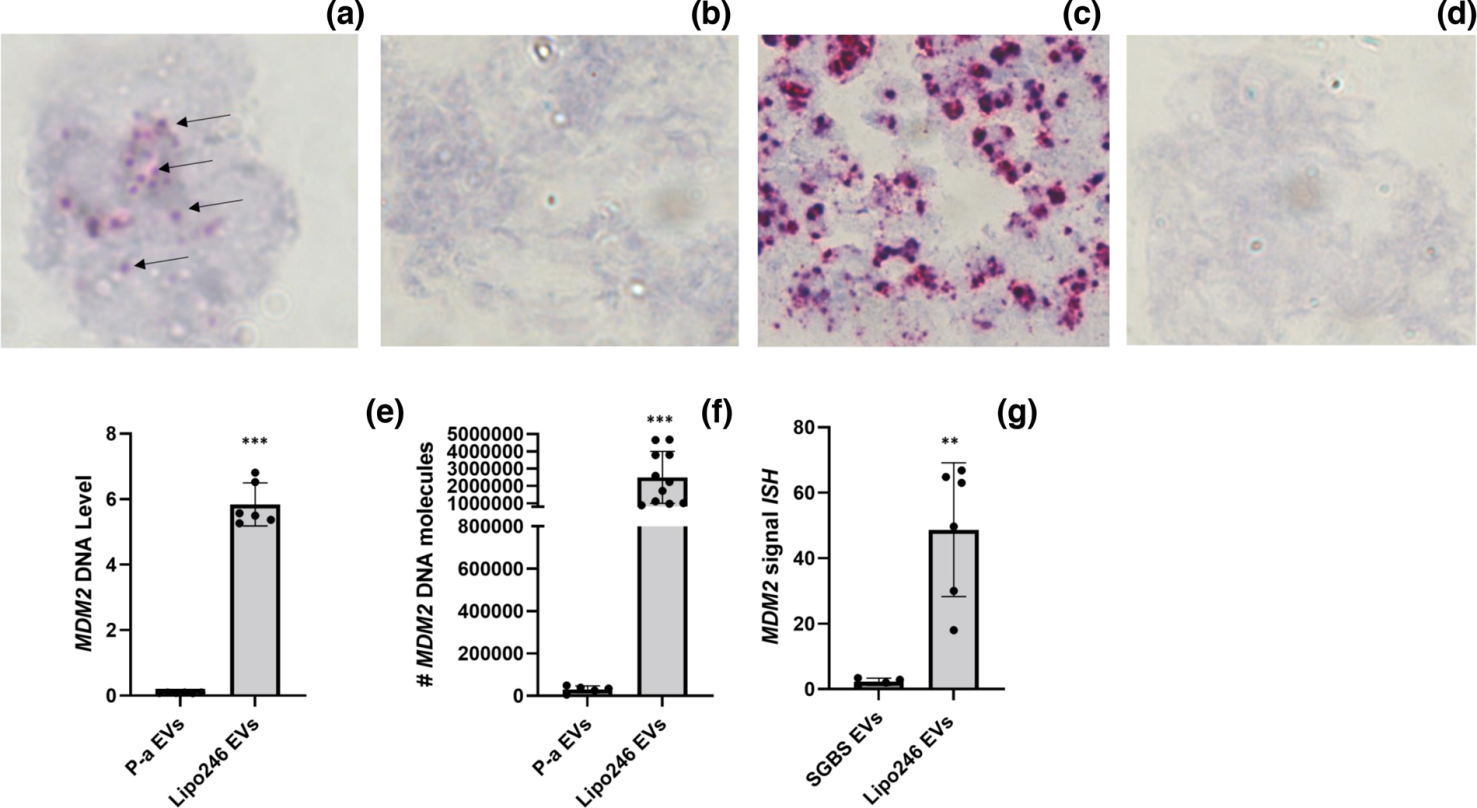
In Situ Hybridization on Cell EVs. ISH for MDM2/CEP12 on EVs isolated from RL cell line (Lipo 246) (a); ISH for MDM2/CEP12 on EVs isolated from control P‐a cell line (SGBS) (b). ISH for MDM2/CEP12 on RL cell line (Lipo246) (c); ISH for MDM2/CEP12 on control P‐a (SGBS) (d), representative images. Level of MDM2 DNA in RL cell versus control P‐a (Q‐PCR) (e); Level of MDM2 DNA in EV isolated from RL cell line (Lipo 246) versus control P‐a cell line (SGBS) (Q‐PCR) (f). ISH signal quantification of MDM2 in EV‐RL cell line (Lipo 246) versus EV‐control P‐a cell line (SGBS) (g). Results are presented as average ± SD. Statistical analyses were performed using an unpaired t test. **, 0.001 ≤ *P* ≤ 0.01; ***, *P* ≤ 0.001

Sections of EVs present in the slides were then probed for MDM2/CEP12. As shown in Figure 5a, after probing we could detect the presence of MDM2 (pink/red dots) in the EVs isolated from Lipo246, suggesting the feasibility of probing a sequence of DNA in EV preparations isolated from cell lines. When probing for MDM2/CEP12 in the EVs isolated from the control P‐a cell line (SGBS), we detected a much lower signal compared to the RL cell line EVs (Figure 5b). We then probed for MDM2/CEP12 in the corresponding cell pellets; as expected (Figure 5c), the signal for MDM2 in the RL cell line was elevated compared to the P‐a control (Figure 5d).

To assess the specificity of the results obtained by ultrasensitive ISH, Q‐PCR was performed on the same preparation of cells analysed by ISH as well as their corresponding EVs. These results demonstrated higher levels of MDM2 in RL cells compared to P‐a controls and higher levels of MDM2 in EVs isolated from RL cells compared to EVs isolated from control cells (Figure 5e, f), as seen in the mean number of MDM2 molecules (P‐a = 0.09 ± sd 0.0095, Lipo246 = 5.839 ± sd 0.66, *p* = 0.000004117; P‐a EVs = 30,625.283 ± sd 16135, Lipo246 EVs = 2,492,774, ± sd 1,501079; *p* = 0.0002846). We then quantified the MDM2 signal in the EV RL cell line and compared this to the EV P‐a cell line signal using ImageJ software (as described in materials and methods). The signal of MDM2 was then normalized to the quantity of EVs present in the slides. This quantity has been determined by IF; representative images are shown in Supplementary Figure 3. As shown in Figure 5g, the normalized MDM2 signal was amplified in the EV isolated from RL cell lines compared to EV MDM2 isolated from P‐a cells (mean MDM2 signal ISH: P‐a EVs = 2.33 ± SD 1.07, RL EVs = 48.72 ± SD 20.43; *p* = 0.00254). Finally, to demonstrate the accuracy of probing EV DNA amplification by ISH in cell EV samples, correlation analysis between MDM2 signals measured by ISH and Q‐PCR has been performed using Spearman's rank correlation test (Spearman's correlation coefficient Rho = 0.72, *p* = 0.024).

### 3.3 In situ hybridization of serum EVs as diagnostic tool

After surgical resection, RL diagnosis is typically confirmed by FISH of the resected tissue. We have previously reported significantly higher levels of MDM2 in EVs isolated from the serum of RL patients compared to normal controls (Q‐PCR; (Casadei et al., 2019)). Therefore, we explored the possibility of using ultrasensitive ISH to assess the presence of MDM2 in patient serum EVs. Serum samples derived from RL patients and healthy donors were processed as reported in materials and methods (see Supplementary Table 2 and Table S3 for patient and controls characteristics). The resultant pellets were divided for EV characterization and for embedding using the system described in materials and methods and summarized in Figure 3. Characterization of EVs isolated from serum samples was determined by nanotrack analysis (Nanosight), TEM and WB for specific protein markers (CD81, CD9, ApoE, Albumin) as shown in Figure 2b, d, f. EVs were then isolated and embedded in paraffin. To verify the presence of EVs in the obtained block, we deparaffinized the blocks, extracted the EVs covered in agarose, and analysed the EVs by TEM. Supplementary Figure 2c shows that EVs are present in the embedded blocks.

**FIGURE 6:**
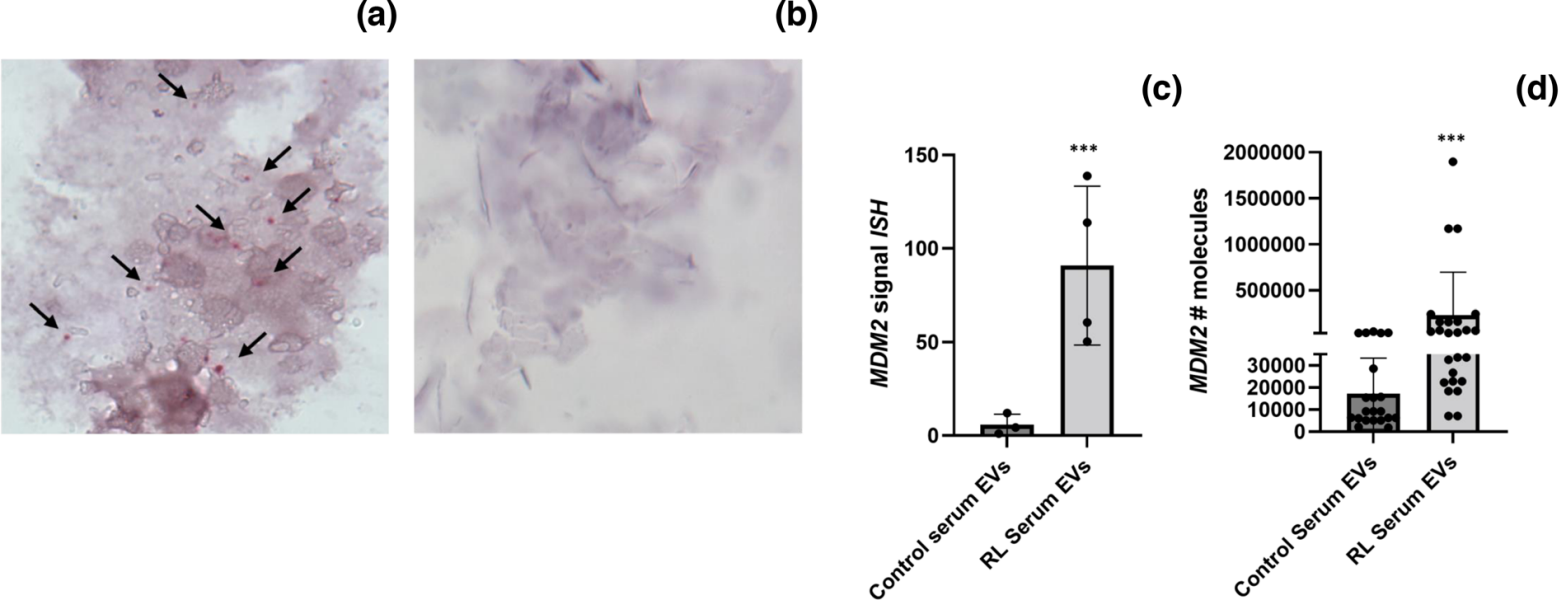
In Situ Hybridization on Serum EVs. ISH for MDM2/CEP12 on EVs isolated from patient serum EVs (a) and on EVs isolated from healthy controls (b), representative images. ISH signal quantification of MDM2 in patient serum EVs versus healthy controls (c). Level of MDM2 DNA in EV isolated from patient serum samples versus healthy controls (Q‐PCR) (d). Results are presented as average ± SD. Statistical analyses were performed using an unpaired t test, and Wilcoxon rank sum exact test was used for Figure 6d. **, 0.001 ≤ *P* ≤ 0.01; ***, *P* ≤ 0.001

Slides were made and ultrasensitive ISH for MDM2/CEP12 was performed. After probing, as shown in Figure 6a, we could detect the presence of MDM2 in the EVs isolated from patient sera, indicating the possibility of probing for a sequence of DNA in the EV preparations isolated from sera. When we probed for MDM2/CEP12 in the EV isolated from normal controls, the MDM2 signal was significantly lower compared to EVs from RL patients (Figure 6b). The MDM2 signal derived from ISH was then quantified using image J and normalized to the quantity of EVs present in the slides. This quantity has been determined by IF; representative images are shown in Supplementary Figure 3. When we quantified the MDM2 signal derived from ISH using ImageJ, we observed significantly higher levels of MDM2 in the EVs of RL serum compared to EV of healthy controls (mean MDM2 signal ISH: control serum EVs = 5.77 ± sd 5.67; RL patient serum EVs = 90.87 ± sd 42.40; *p* = 0.00028, Figure 6c). Finally, Q‐PCR of the EVs isolated from a second cohort of RL serum (*N* = 19) and control (*N* = 15) was also performed, confirming the significantly higher level of MDM2 in RL serum EVs compared to normal controls (mean MDM2 number of molecules: control serum EVs = 16,329 ± 16,395, patient serum EVs = 230,149 ± 480,313; *p* = 6.29E‐05, Figure 6d). There were insufficient serum samples to perform Spearman's correlation (the amount of serum available for each patient was not enough to perform Q‐PCR and ISH on the same patient cohort).

## 4 DISCUSSION

Interest in EVs and their cargoes has increased due to a new appreciation of their role in communication between tumour and microenvironment and involvement in cancer progression. Utilizing imaging techniques has been recognized as a next step to elucidate the function of EVs in normal and diseased states (Verweij et al., 2021). Moreover, faster and more convenient methods are required to validate EV cargoes as cancer biomarkers (Yu et al., 2022). The challenge of visualizing EV cargoes is still a relevant yet unresolved issue in the literature of the filed. After showing the successful isolation of EVs from RL tissues, we demonstrate an ultrasensitive ISH methodology for visualization of specific DNA target, in EVs isolated from tissue, CM and serum when embedded in paraffin agarose blocks.

Most information regarding the roles of EVs and their cargoes in disease has been based on studies on EVs isolated from cells in culture. However, such in vitro systems have limitations; for example, the possibility that high numbers of culture passages may result in cell preparations no longer accurately reflecting the disease of interest (Ramirez et al., 2018), a problem shared with EVs isolated from such long term cultured cell lines (Crescitelli et al., 2021). In the context of cancer, interstitial EVs may be critical in modulating the tumour microenvironment for subsequent cancer cell seeding and metastatic growth (Hurwitz et al., 2019); characterization of EV cargoes within the primary tumour tissue milieu could be very important in this context. However, isolation and sufficient yield of EVs from tissues (where EVs are trapped within the cellular structures and extracellular matrix components) is difficult due to the challenge of tissue dissociation without cellular or EV damage; variation in EV isolation protocols may lead to heterogeneous and irreproducible results (Lugini et al., 2016; Xu et al., 2018). Consequently, establishing new methods for EVs isolation from tissues remain an important and unresolved issue. In this study, we optimized the methodology for the successful isolation of EVs from liposarcoma tissues.

DNA as EV cargo, has been implicated in various disease processes as a mediator of physiological functions (Malkin & Bratman, 2020); Interestingly, cells with specific mutations in their genomic DNA (gDNA) release EVs containing DNA that harbour identical mutations (Jabalee et al., 2018). It is also very intriguing that EV DNA, when transferred to other cells, may modify the recipient cells, leading to increased gDNA‐coding mRNA, protein expression, and consequent altered function in these recipient cells (Cai et al., 2013). However, several questions regarding biogenesis, structure, localization and function of EV DNA remain unanswered (Malkin & Bratman, 2020). Little is known regarding how DNA is sorted into EVs, whether DNA in the EVs may be single‐or double stranded, whether such DNA resides within the lumen or on the surface of EVs, and which subpopulation of EVs (i.e., small or large EVs) are associated with EV DNA cargoes (Malkin & Bratman, 2020). Although many studies have reported the possibility that EV cargoes may be transferred to recipient cells, the underlying mechanisms are not clearly understood, especially when the cargo is functional DNA. The methodology proposed here permits visualization of specific DNA targets, facilitating study of amplification, aberration and other alterations of DNA present as cargo in EVs from tissue, CM, and serum.

Readily retrievable by liquid biopsy, EV DNA cargo has been recently considered as a promising biomarker of tumour presence and complexity (Amintas et al., 2020; García‐Silva et al., 2020). Cell‐free DNA (cfDNA) and EV DNA have both been studied as possible diagnostic confirmations for many types of cancers. Compared to diagnostic methods based on cfDNA, EV DNA confers several advantages, including its relatively greater abundance as packaged EV DNA cargoes, the increased stability of EVs in the circulation, high sensitivity and specificity of EV DNA mutational frequency, and the comparative consistency of EV cargo accuracy for diagnosis and prognosis prediction purposes (Allenson et al., 2017; García‐Silva et al., 2020; Hoshino et al., 2015; Wan et al., 2018; Xu et al., 2016; Zhou et al., 2020; Zocco et al., 2020). Several studies have demonstrated that EV DNA allows detection of mutations that accurately reflect the tumour of origin mutational state (Kahlert et al., 2014; Kunz et al., 2019; Zocco et al., 2020). Moreover, EV DNA has the additional potential of being retrievable from multiple body fluids of differing composition. A limitation in the use of EV DNA testing is the lack of standardized isolation and normalization methodologies (García‐Silva et al., 2020; Kahlert, 2019; Ramirez et al., 2018; Royo et al., 2020), making their application as potential biomarkers challenging at this time (Zhou et al., 2020). FISH is traditionally used to determine gene amplification, rearrangements and/or fusions in resected tumour tissues. As alternative, Q‐PCR is also used for a similar purpose in the clinic. It has been shown that FISH of resected tissue and Q‐PCR, when used in combination, increases the diagnostic verification of many diseases (Haidary et al., 2019; Nistor et al., 2006). Likewise, it may be that the method proposed here (DNAscope on embedded EVs) in combination with Q‐PCR could overcome diagnostic limitations of solo EV Q‐PCR. This is especially pertinent in light of new microfluidic devices being developed by us and others that may facilitate EV DNA evaluation applications as future routine clinical diagnostics (Casadei et al., 2021).

In situ hybridization has been used for identifying specific genes in specific cell populations; but traditional ISH methods are not sensitive enough to localize a single molecule in individual cells. Recently, with the development of ‘tree’ based ISH techniques, it became possible to increase sensitivity and localize single molecules (Wang et al., 2012). Scope methodologies (DNA/RNAscope) have been shown to have high sensitivity and specificity, allowing better reproducibility for gene biomarker validation (Morley‐Bunker et al., 2021). An appealing feature of scope methodologies is that DNA/mRNA expression can be quantified at a single cell level with the use of image analysis methods (image J) and brightfield microscopy. Studies comparing scope technologies and Q‐PCR have been previously performed, suggesting that this methodology performs at a level similar to Q‐PCR (Morley‐Bunker et al., 2021). Morely‐Bunker et al. suggested that, using Image J to analyse the images obtained by Scope methodologies, the limits of agreement was 0.11 a.u. The versatility of this method and ease of use makes this approach very attractive for EV DNA studies as an alternative to other more complicated and expensive techniques requiring laborious optimization. In this work, when applied to embedded EVs, the coefficient of correlation between Q‐PCR and signals of DNAscope was close to 1, confirming the accuracy of ISH for probing EV DNA amplification.

Detection of EV cargoes remain challenging; faster and more convenient methods are required to validate EV cargoes. Moreover, most recently developed methods for EV cargo visualization have rarely been applied to DNA as cargo. Single‐molecular localization microscopy (SMLM) techniques have recently been applied to EV nucleic acid cargoes. An application note (https://oni.bio/oni‐articles) suggested the possibility of visualizing generic EV DNA cargo (not a specific gene) by direct stochastic optical reconstruction microscopy (dSTORM, ONI). This elegant technique, available only a few centres, requires tedious set up and troubleshooting that hinders ease of applicability, which may explain the small number of publications to date that use this technology (Brunner et al., 2021; Mcnamara et al., 2022). Recently Yu et al. summarized conventional and novel technologies for EVs isolation, characterization, and content detection, particularly in the context of EVs in liquid biopsy. WB and ELISA assay have been recognized as regular approaches to detect EV proteins, but they can be complex and can have low sensitivity. Accordingly, a new series of protein detection methods (colorimetric, fluorescence, electrochemical etc.) have been now developed (Yu et al., 2022). Conventional methods for nucleic acids analysis include q‐PCR together with microarray and NGS. Yu et al. also catalogued efforts to develop highly sensitive and convenient methods for EV nucleic acid detection. These methods described in that review have been used to detect mRNA and/or miRNA, but not DNA; each of them have been tested in CM or serum or urine or tissues, but not in all the systems. Of the various methods ddPCR, only applied to detect miRNA and mRNA, has the disadvantage of being high‐cost, limited through‐put and complex operation; molecular beacons have been only applied to detect miRNA (not DNA), have high‐cost and limited through‐put; DNA tetrahedron probe has high costs; SPR detection and Single Vesicle Analysis both have nonspecific adsorption. Molecular beacon (MB) are probe molecules that, in a manner similar to TaqMan probes, can emit fluorescence upon hybridization to a complementary target sequence (Tan et al., 2000). The presence of EV miRNA targets is measured by fluorescence intensity. They are proposed in some reports for in situ detection of EV miRNAs (in serum or plasma or urine (Chen et al., 2021; Cho et al., 2019; Lee et al., 2016, 2015, 2018; Oliveira et al., 2020)). While MB are supposed to be delivered inside the EVs thereby probing EV cargos (such as miRNAs), this methodology does not allow contemporary imagining of EVs and targets; molecular beacons have been used to probe for specific EV miRNAs (miR‐21) in breast cancer CM and serum (Lee et al., 2015) as well as in urine samples of prostate cancer patients (Lee et al., 2018). However, this method is problematic in that the molecular beacons may not be delivered into the EVs in equal amount, causing signal deviation and failure to accurately quantify the content of EV miRNAs. Wang et al. proposed a methodology for the detection of serum EV miRNAs integrating a multiple recognition sequence into a probe (Wang et al., 2020). This system was tested using miR‐27a, miR‐21 and miR‐375 in CM of breast cancer cells. However, even after these discoveries, probing for RNA remained a challenge. Therefore, in 2021 Fang et al. developed a system to target mRNA using a target‐triggered self‐assembled DNA tree for amplified analysis of mRNA in intact living cells (Fang et al., 2021). Ahmed et al. (2018) used immunogold labelled electron microscopy to detect Epstein–Barr virus‐encoded small RNAs on ultra‐thin sections of EBV infected and non‐infected B‐cell lines. Notably, in 2019 Gupta et al. developed a methodology which enables the detection of EVs in tissues (in the vitreous humour of the eye and in mouse mammary carcinoma) using non‐reversible tissue fixation; this methodology allows the visualization of EVs through confocal microscopy. They also were able to mark general RNA and DNA with propidium Iodide (PI), but not specific DNA/RNA targets (Gupta et al., 2019) in EV tissues.

In contrast to the previously described work, we present here a new method in which EVs are embedded in agarose and paraffin, upon isolation from tissues, cell lines and sera. We also show the possibility of detecting specific DNA targets in slides of these paraffin embedded EVs. Using this method, we detected DNA; the samples in which DNA was detected were neither fresh pellets nor solutions or EVs fixed and stable for only a limited time. Instead, this new method uses EVs embedded in paraffin, hence they are stable, shelf storable, and available for analysing over many future years. The new methodology is neither complicated nor expensive; it does not require time consuming optimization of any parameters. Moreover, the complicated process of probes and fluorescent tag assemblage, is avoided because custom probes are provided by DNAscope. It also does not require particular expertise, since it uses readily available traditional methodologies, for example, paraffin blocks, cut onto slides, ISH, and brightfield microscopy. It can be applied to CM, serum and EVs isolated from tissues.

The main genetic characteristic of liposarcoma is amplification of the MDM2 gene located on chromosome 12q13‐15, detectable by FISH. In our previous work, we have shown that MDM2 DNA is amplified in RL EVs (Casadei et al., 2019). We now demonstrate that MDM2 DNA can be visually detected in EVs by an ultrasensitive ISH using embedded EVs. MDM2 is amplified in more than 40 different types of malignancies, including sarcomas, other solid tumours, and leukaemias (Casadei et al., 2019; Rayburn et al., 2005); therefore, with selection of appropriate probes, researchers may be able to identify DNA target sequences and specific DNA alterations in tumour tissue‐associated‐EVs, opening the possibility for more extensive study of EVs in relation to a wide array of diseases.

## 5 CONCLUSION

In conclusion, our findings show that EVs isolated from tissues, cell lines and sera can be embedded and used as substrate to detect specific sequences of DNA through ultrasensitive in situ hybridization, using detection of MDM2 amplification in embedded EVs isolated from liposarcoma tissues, cell lines and patient sera as an example. This methodology may allow the visualization of DNA targets in a simple fashion, without time consuming optimization steps and particular expertise. Formalin‐fixed paraffin‐embedded (FFPE) blocks of EVs will allow long‐term preservation of the samples, permitting further studies. Furthermore, modification of probes may enable researchers to detect targets and specific DNA alterations directly in tumour EVs, thereby facilitating detection, diagnosis, and improved understanding of tumour biology relevant to many cancer types.

## CONFLICT OF INTEREST

The authors report no conflict of interest.

## Supporting information

Supporting Information
