# Supplementary figures and images for: In situ hybridization to detect DNA amplification in extracellular vesicles
Source: J Extracell Vesicles. 2022 Aug 31;11(9):e12251. doi: 10.1002/jev2.12251 (PMC9428764; doi:10.1002/jev2.12251)

## Slide 1
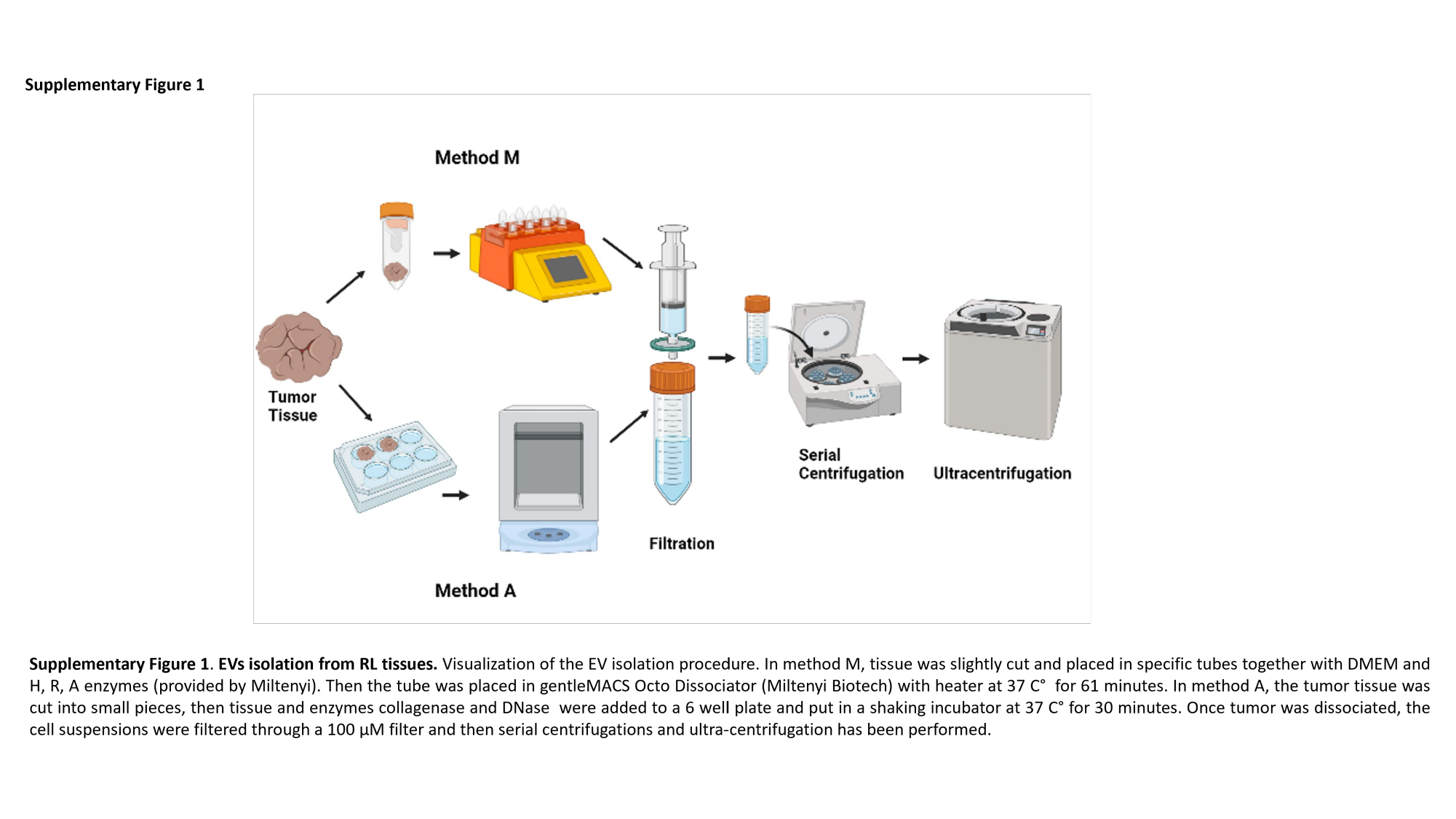

## Slide 2
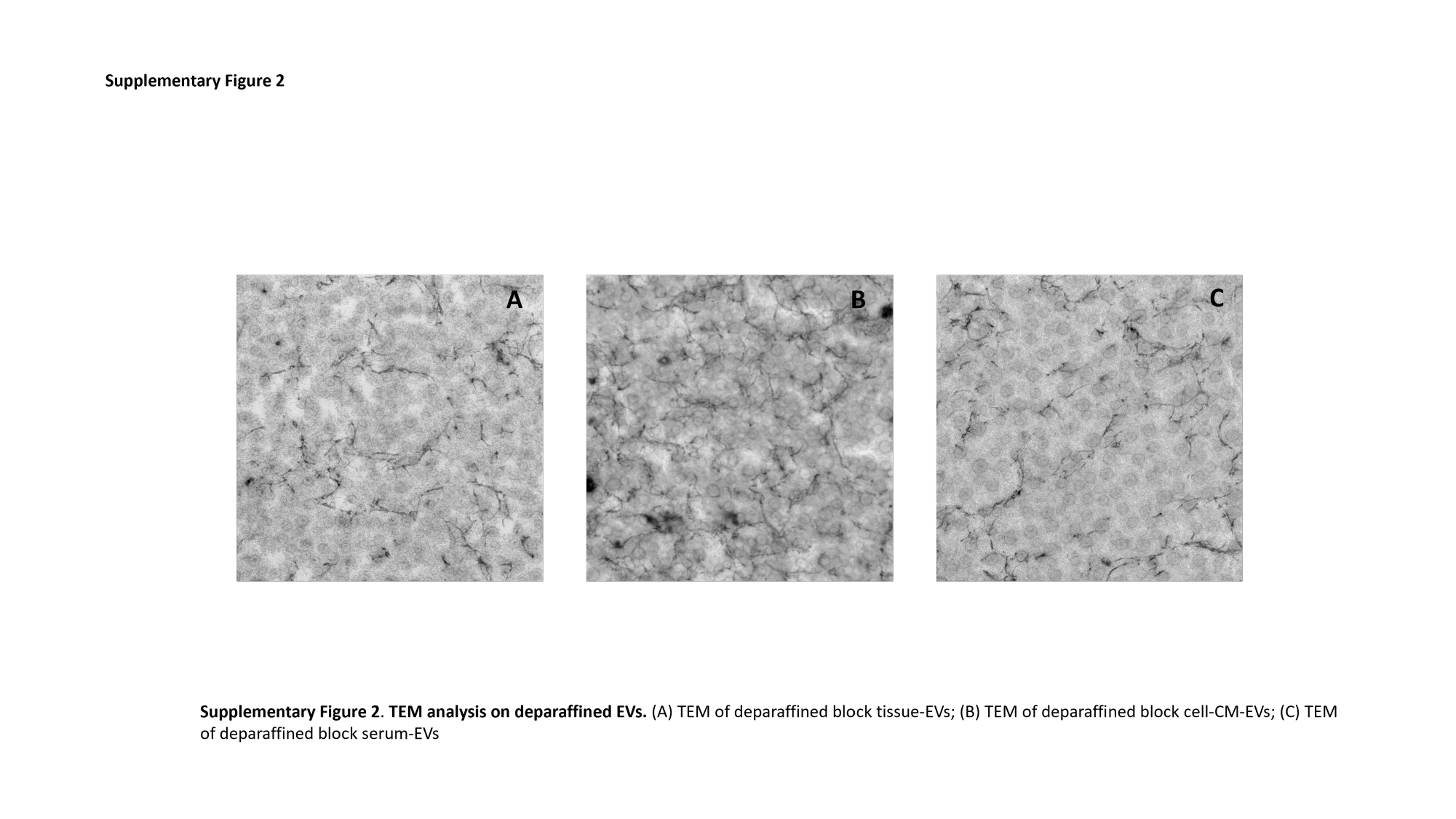

## Slide 3
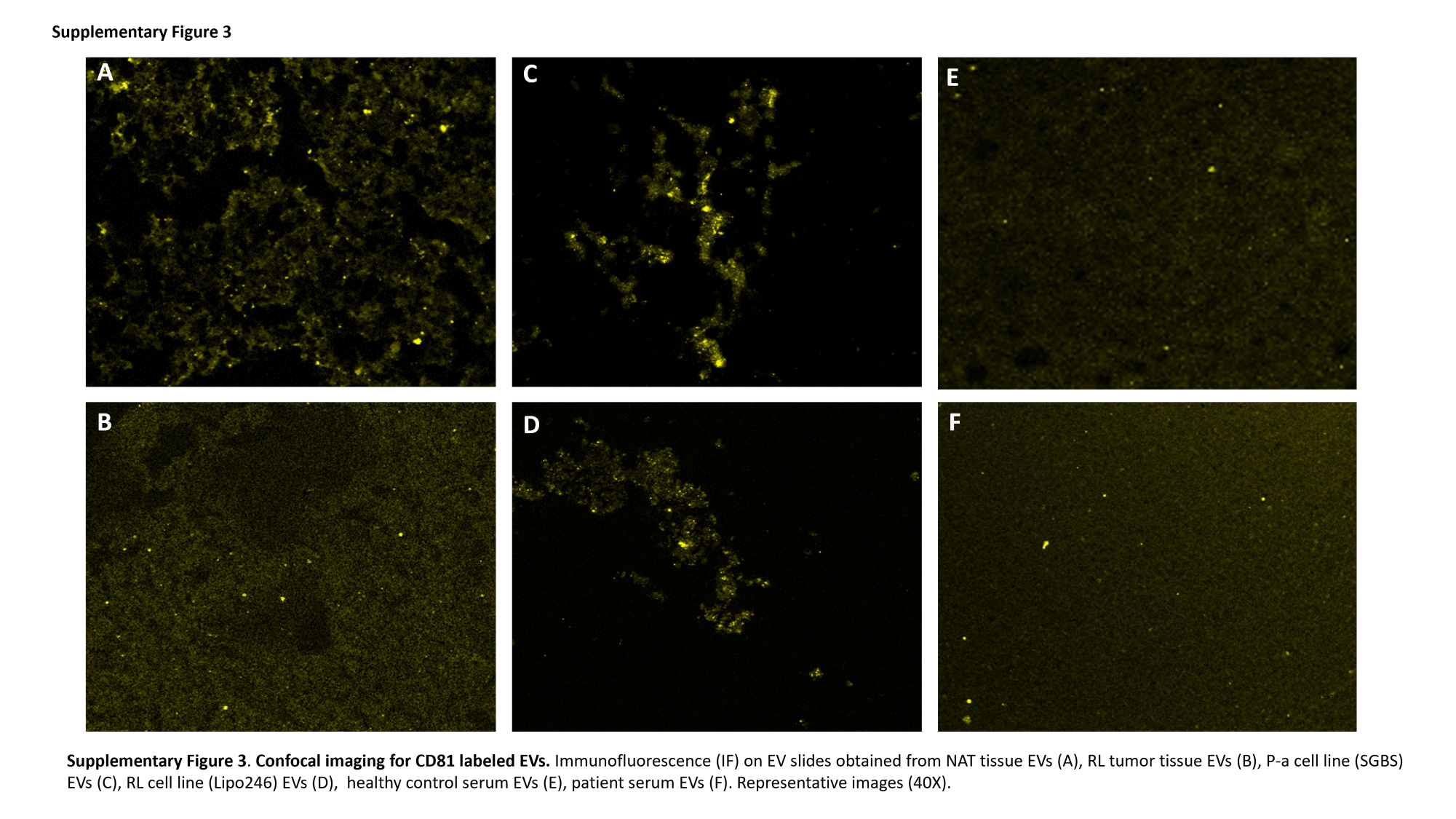

## Slide 4
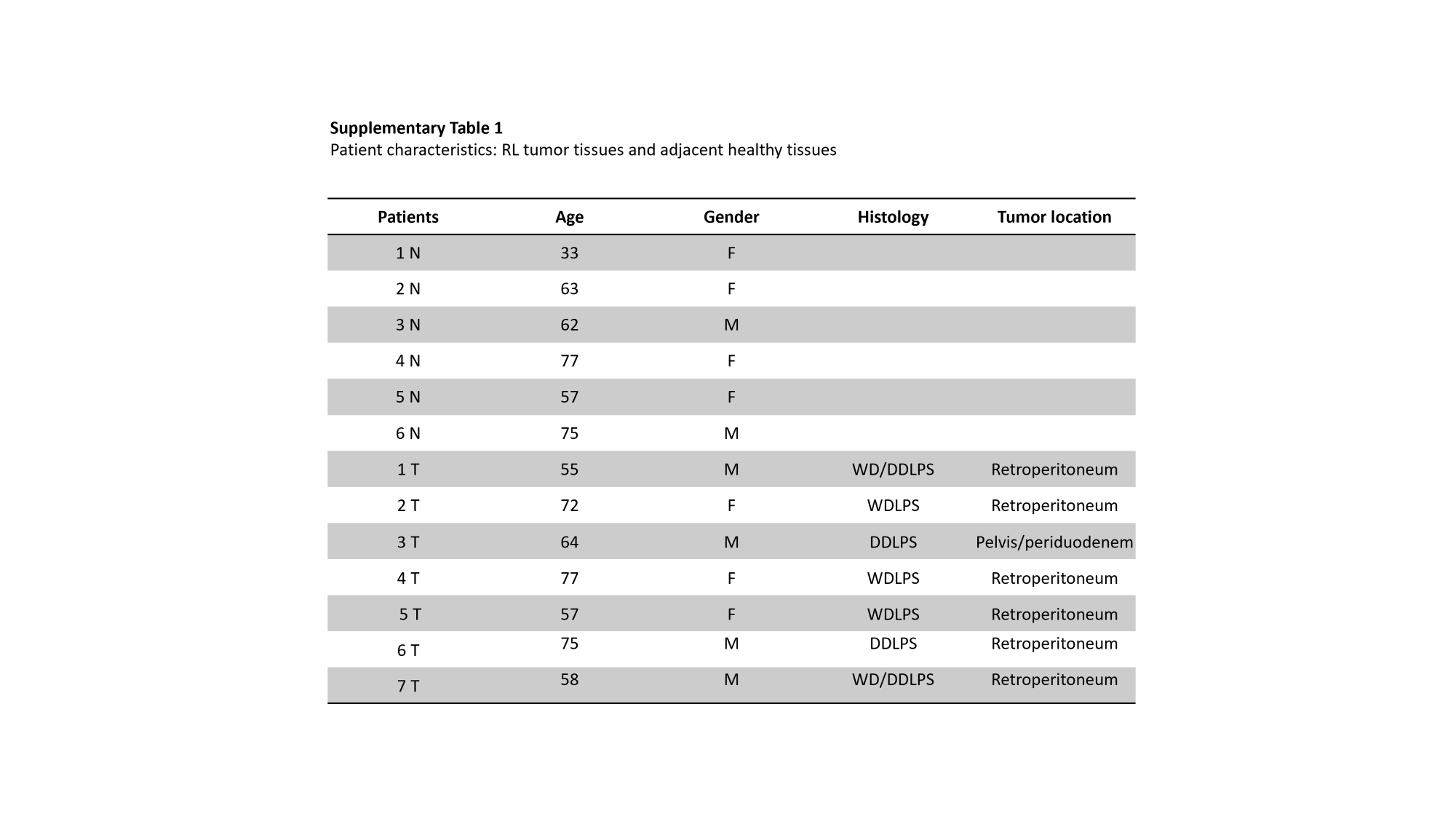

## Slide 5
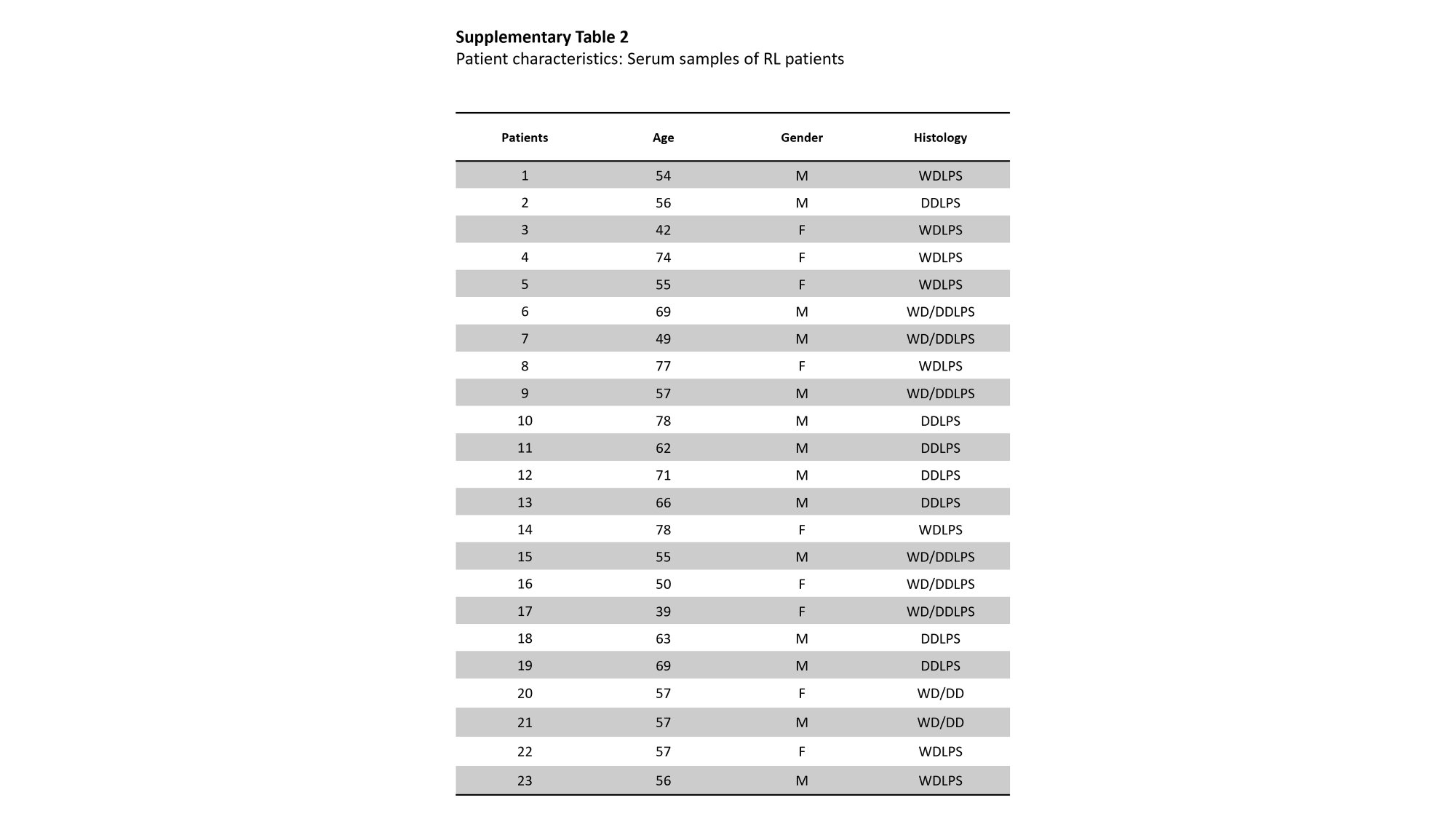

## Slide 6
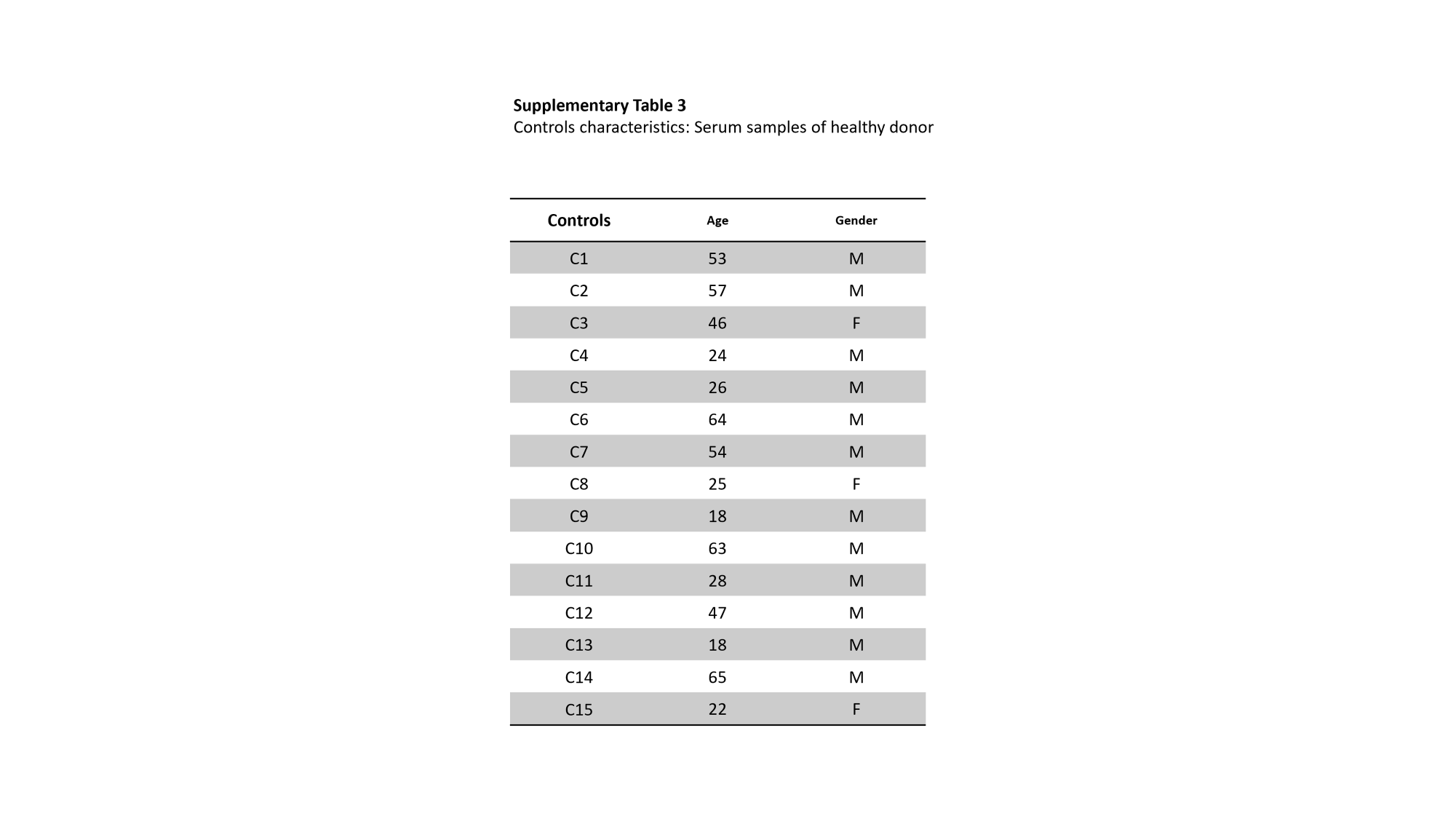

## Slide 7
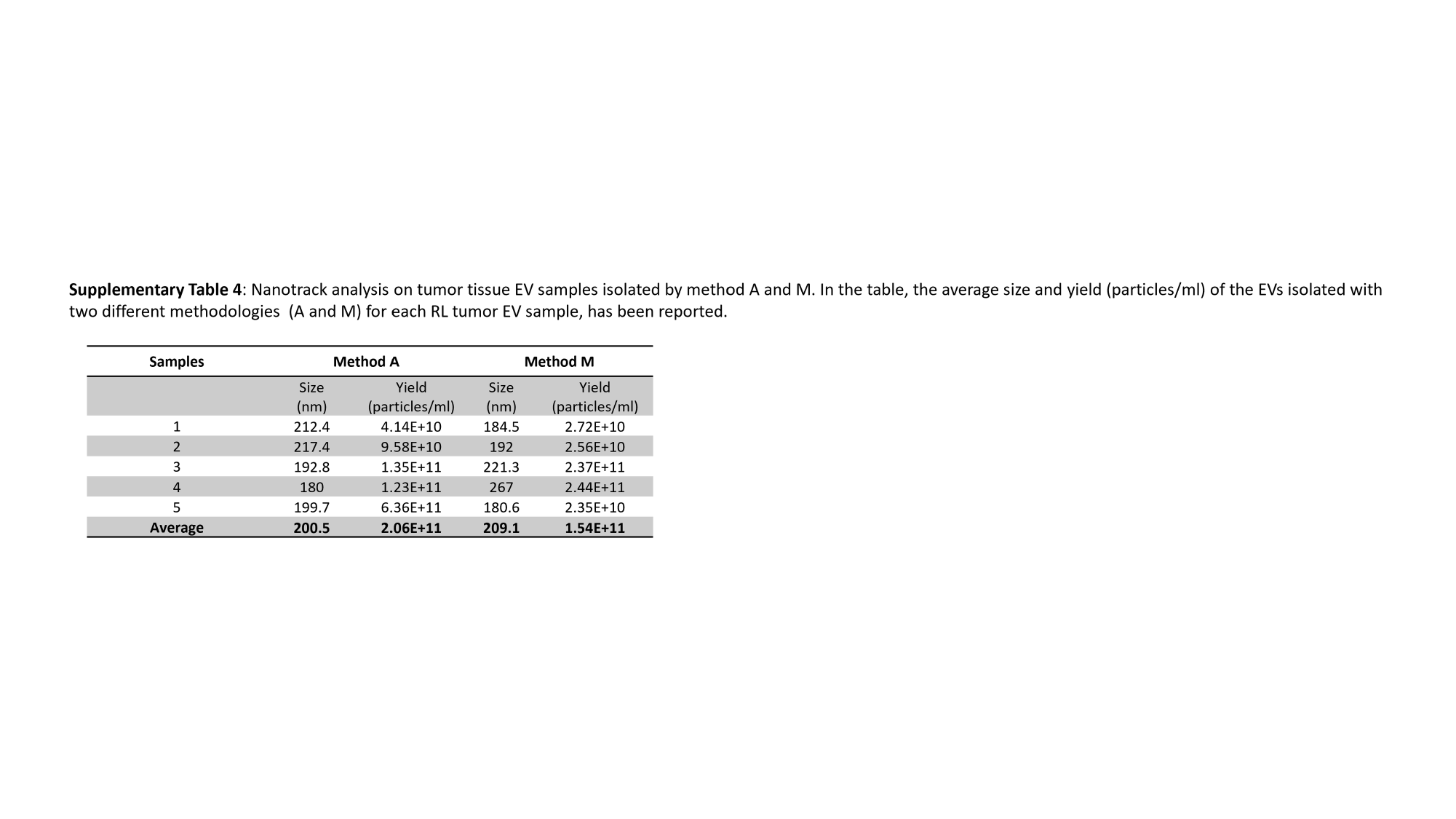

Supplement: Supplementary file 1 — Supporting Information [file JEV2-11-e12251-s001.pptx]
